# Supplementary material for: Angelica sinensis polysaccharide as potential protectants against recurrent spontaneous abortion: focus on autophagy regulation
Source: Front Med (Lausanne). 2025 Jan 15;12:1522503. doi: 10.3389/fmed.2025.1522503 (PMC11774876; doi:10.3389/fmed.2025.1522503)
Supplement: Supplementary material S1 — The main instruments used during the LC-MS process, along with their models/specifications and manufacturers. [file Supplementary_file_1.zip › Metabolomics sequencing data FC1.2/8.代谢通路分析/Exposure_Ctrl/all/KEGG/mmu04071.html]

KEGG PATHWAY: Sphingolipid signaling pathway - Mus musculus (house mouse)


# Sphingolipid signaling pathway - Mus musculus (house mouse)


[
Pathway menu
| Organism menu
| Pathway entry
| Show description
| Download
| Help
]

Sphingomyelin (SM) and its metabolic products are now known to have second messenger functions in a variety of cellular signaling pathways. Particularly, the sphingolipid metabolites, ceramide (Cer) and sphingosine-1-phosphate (S1P), have emerged as a new class of potent bioactive molecules. Ceramide can be generated de novo or by hydrolysis of membrane sphingomyelin by sphingomyelinase (SMase). Ceramide is subsequently metabolized by ceramidase to generate sphingosine (Sph) which in turn produces S1P through phosphorylation by sphingosine kinases 1 and 2 (SphK1, 2). Both ceramide and S1P regulate cellular responses to stress, with generally opposing effects. S1P functions as a growth and survival factor, acting as a ligand for a family of G protein-coupled receptors, whereas ceramide activates intrinsic and extrinsic apoptotic pathways through receptor-independent mechanisms.


##### Option

Scale:


100%

Image resolution:


 High

##### Search

##### ID search

##### Color


KGML

Image (png) file 1x
